# Coronin 1B Controls Endothelial Actin Dynamics at Cell–Cell Junctions and Is Required for Endothelial Network Assembly

**DOI:** 10.3389/fcell.2020.00708

**Published:** 2020-07-31

**Authors:** Ann-Cathrin Werner, Ludwig T. Weckbach, Melanie Salvermoser, Bettina Pitter, Jiahui Cao, Daniela Maier-Begandt, Ignasi Forné, Hans-Joachim Schnittler, Barbara Walzog, Eloi Montanez

**Affiliations:** ^1^Institute of Cardiovascular Physiology and Pathophysiology, Biomedical Center, LMU Munich, Munich, Germany; ^2^Walter Brendel Center of Experimental Medicine, University Hospital, LMU Munich, Munich, Germany; ^3^Medizinische Klinik I, Klinikum Großhadern, Munich, Germany; ^4^Institute of Anatomy and Vascular Biology, Westfälische Wilhelms-Universität Münster, Münster, Germany; ^5^Protein Analysis Unit, Biomedical Center, LMU Munich, Munich, Germany; ^6^Department of Physiological Sciences, Faculty of Medicine and Health Sciences, University of Barcelona and IDIBELL, Barcelona, Spain

**Keywords:** endothelial cells, cell–cell junction, actin, tube formation, coronin 1B

## Abstract

Development and homeostasis of blood vessels critically depend on the regulation of endothelial cell–cell junctions. VE-cadherin (VEcad)-based cell–cell junctions are connected to the actin cytoskeleton and regulated by actin-binding proteins. Coronin 1B (Coro1B) is an actin binding protein that controls actin networks at classical lamellipodia. The role of Coro1B in endothelial cells (ECs) is not fully understood and investigated in this study. Here, we demonstrate that Coro1B is a novel component and regulator of cell–cell junctions in ECs. Immunofluorescence studies show that Coro1B colocalizes with VEcad at cell–cell junctions in monolayers of ECs. Live-cell imaging reveals that Coro1B is recruited to, and operated at actin-driven membrane protrusions at cell–cell junctions. Coro1B is recruited to cell–cell junctions via a mechanism that requires the relaxation of the actomyosin cytoskeleton. By analyzing the Coro1B interactome, we identify integrin-linked kinase (ILK) as new Coro1B-associated protein. Coro1B colocalizes with α-parvin, an interactor of ILK, at the leading edge of lamellipodia protrusions. Functional experiments reveal that depletion of Coro1B causes defects in the actin cytoskeleton and cell–cell junctions. Finally, in matrigel tube network assays, depletion of Coro1B results in reduced network complexity, tube number and tube length. Together, our findings point toward a critical role for Coro1B in the dynamic remodeling of endothelial cell–cell junctions and the assembly of endothelial networks.

## Introduction

The formation of new blood vessels through angiogenesis involves endothelial cell (EC) adhesion, migration and proliferation and is critical for embryo development and tissue regeneration (Potente et al., 2011). Angiogenesis relies on the dynamic rearrangement of VE-cadherin (VEcad)-mediated cell–cell junctions (Giannotta et al., 2013; Cao and Schnittler, 2019). Endothelial cell–cell junctions are also crucial for vessel permeability, vessel stability and vascular integrity (Dejana et al., 2009). Consequently, perturbations in cell–cell junction organization and function result in developmental defects and vascular pathologies including chronic inflammation, edema and atherosclerosis (Weis et al., 2004; Dejana et al., 2009). In addition to VEcad, ECs also express N-cadherin (Ncad) (Lampugnani et al., 1992), which has a dispersed distribution along cell membranes (Navarro et al., 1998; Kruse et al., 2019). Both cadherins contribute to establishing the endothelial barrier, but Ncad plays a key role in recruiting pericytes during angiogenesis (Gerhardt et al., 2000; Luo and Radice, 2005; Tillet et al., 2005). Although many aspects of blood vessel formation and homeostasis depend on cell–cell junctions, the molecular mechanisms that regulate their dynamic rearrangement are not fully understood.

The VEcad-catenin complex, which constitute the molecular basis of the adherens junctions, is connected to the actin cytoskeleton and its function is regulated by signal transduction, cytoskeletal contraction, and actin-driven plasma membrane protrusions (Dejana and Vestweber, 2013; Giannotta et al., 2013; Cao et al., 2017; Paatero et al., 2018; Cao and Schnittler, 2019). Endothelial adherens junctions are highly dynamic and therefore require constant VEcad rearrangement (Bentley et al., 2014). Junction-associated intermitted lamellipodia (JAIL) are small actin-driven protrusions at cell–cell junctions controlled by the actin related protein 2/3 (Arp2/3)-complex that contribute to the regulation of cell–cell junctions (Abu et al., 2014). JAIL driving VEcad dynamics within the cell–cell junction is critical for monolayer integrity, cell migration and angiogenesis (Abu et al., 2014; Fraccaroli et al., 2015; Cao et al., 2017). JAIL develop from branched actin filament and protrude across a small area of the apical membrane of the adjacent cell. In the overlapping area, VEcad plaques emerge due to trans-interactions between VEcad molecules (Abu et al., 2014). JAIL formation is terminated by the dissociation of the Arp2/3 complex from actin filaments. Actin disassembly leads to translocation of clustered VEcad molecules from the VEcad plaque to the junction resulting in the formation of a new VEcad adhesion site. JAIL formation depends on the relative concentration of VEcad at the cell–cell junction, however, the molecular mechanisms regulating JAIL during vessel development are not completely understood.

Actin-binding proteins regulate actin cytoskeleton dynamics thereby controlling the remodeling of endothelial cell–cell junctions, cell migration and vessel integrity (Pollard et al., 2000; Edwards et al., 2014). Several actin-binding proteins including EPLIN and α-parvin (α-pv) colocalize with and control JAIL formation (Fraccaroli et al., 2015; Taha et al., 2019). Parvins are a family of adaptor proteins that localize to focal complexes and focal adhesions, and facilitate the interaction of integrins with the actin cytoskeleton (Olski et al., 2001; Legate et al., 2006). Coronins are a family of actin-binding proteins that regulate actin polymerization via binding to and inhibiting the Arp2/3 complex (Cai et al., 2008; Chan et al., 2011; Howell et al., 2015). Type I coronins, such as coronin 1B (Coro1B), localize to the leading edge of migrating cells where they regulate actin dynamics in the lamellipodia via both Arp2/3 complex and cofilin-mediated pathways (Mishima and Nishida, 1999; Chan et al., 2011). Coro1B also fine-tunes ROCK-signaling pathway to regulate myosin activity (Rana and Worthylake, 2012; Priya et al., 2016). As such, type I coronins regulate actin-dependent processes including cell migration (Foger et al., 2006; Cai et al., 2007; Samarin et al., 2010; Howell et al., 2015). In epithelial cells, Coro1B controls actin cytoskeleton reorganization and cell–cell junction stability through RhoA signaling (Michael et al., 2016; Priya et al., 2016). Coro1B is expressed in ECs suggesting that it might regulate actin dynamics of blood vessels (Usatyuk et al., 2013; Kim et al., 2016), however, its role in ECs is not fully understood. In the current study we identify Coro1B as a new regulator of JAIL formation, cell–cell junction and the assembly of endothelial network *in vitro*.

## Materials and Methods

### Cell Culture

Mouse embryonic ECs were isolated as previously described (Fraccaroli et al., 2015) and cultured in EC growth medium (Promocell). Human umbilical vein endothelial cells (HUVECs) (Pelobiotech) were cultured in EC medium (Promocell). Human dermal microvascular endothelial cells (HMECs) (American Type Culture Collection) were cultured in Dulbecco’s Modified Eagle Medium (Thermo Fisher Scientific) supplemented with 10% EC growth medium (Promocell), 10% fetal calf serum (FCS) and 1% Penicillin/Streptomycin.

### Antibodies and Reagents

The following antibodies and reagents were used for the analyses: the rabbit antibody against Coronin 1B (Sigma-Aldrich); mouse antibody against CD144 (VE-cadherin) (eBioscience), rabbit antibody against integrin-linked kinase (ILK) (Cell Signaling Technology) and rabbit antibody against α-parvin (Cell Signaling Technology). For secondary detection, species-specific Alexa Fluor-coupled secondary antibodies (Invitrogen) were used. Filamentous actin (F-actin) was visualized with Phalloidin Alexa-633 (Invitrogen). HUVEC cells were treated with thrombin (Sigma-Aldrich) and Y-27632 (Merck Millipore).

### Immunostaining

For immunostaining, cells were seeded on glass coverslips coated with 0.15% gelatin. In indicated experiments, cells were treated with 0.2 U/ml thrombin or 10 μM Y-27632 for 10 min. Cells were fixed in 4% paraformaldehyde for 10 min, permeabilized with 0.1% Triton X-100 for 30 min and incubated with blocking solution (1% bovine serum albumin, 0.1% Triton X-100 in PBS) for 1 h at room temperature. Cells were exposed to primary antibodies overnight at 4°C. After washing three times with 0.1% Triton X-100 in PBS for 15 min, secondary antibodies were applied for 1 h at RT. After washing three times with PBS for 15 min, cells were embedded in Fluoromount (Southern Biotech) and analyzed with a Leica SP8X WLL upright confocal microscope (Leica).

### Cloning, Gene Transduction, Transfection, and Live-Cell Imaging

To generate the Coro1B-GFP fusion protein, the Coro1B_pMK-RQ plasmid (Invitrogen) was amplified with the primers 5′-GCATAAGCTTATGTCCTTCCG-3′ and 5′-GTGAAAGGAAGGCCCATGA-3′ and the coding region for Coro1B was cloned into the vector pEGFP-N1 (Clontech) with using *Sac*II and *Hin*dIII and T4-Ligase (New England Biolabs). Recombinant lentiviral vectors carrying Lifeact-mCherry and VEcad-mCherry were kindly provided by Hans Schnittler. HUVECs were transduced with Lifeact-mCherry or VEcad-mCherry by incubation with viral particles resuspended in EC growth medium containing 3% poly-vinyl-pyrrolidone for 1 h. Afterward cells were transfected with Coro1B-GFP by using the MATra-A reagent (Promocell) and a magnet plate (Promocell) according to manufacturer’s protocol. Cells were further cultured and after 48–72 h time-lapse recording with 63× objective at a spinning-disk confocal microscope (Carl Zeiss) at 37°C and 5% CO_2_ was performed. The coding region for Coro1B-GFP was amplified with the primers 5′-TCG GCGCGCCACGCGTATGTCCTTCCGCAAAGTGG-3′ and 5′-AATGTTAACGACCGTTTACTTTACTTGTACAGCTCGTCC AT-3′ and by using *Mlu*I and *Age*I restriction sites with In-Fusion^®^ HD Cloning Kit (Clontech) cloned into the lentiviral vector pLV-CMV-MCS-IRES-PURO-SIN. The coding region of GFP was amplified with the primers 5′-TCGGCGCGCCACGCGTATGGTGAGCAAGGGCGAGGAGC-3′ and 5′-CGGCATGGACGAGCTGTACAAGTAAACCGGTCG TTAACATT-3′ and subcloned into pLV-CMV-MCS-IRES-PURO-SIN using *Mlu*I and *Age*I restriction sites with In-Fusion^®^ HD Cloning Kit (Clontech). Lentivirus encoding for Coro1B-GFP or GFP as control and a puromycin resistance was produced by transfection of human embryonic kidney 293T cells with the generated pLV-CMV-Coro1B-EGFP-IRES-PURO-SIN vector or pLV-CMV-EGFP-IRES-PURO-SIN vector, respectively, the envelope vector pCMV-VSV-G (Addgene) and the packaging vector pCMV-ΔR8.91 (Addgene) using Lipofectamin 2000 (Thermo Fisher Scientific) according to the manufacturer’s protocol. Supernatant was collected after 48 h and filtered using 0.45 μm filters. HMECs were transduced by incubating with viral particles resuspended in Dulbecco’s Modified Eagle Medium containing 10% EC growth medium, 10% FCS and 1% Penicillin/Streptomycin for 24 h. To generate cell lines stably expressing Coro1B-GFP and GFP, respectively, 72 h post transduction 10 μg/mL puromycin was added to the culture medium.

### Co-immunoprecipitation and Western Blot

Stably Coro1B-GFP or GFP expressing HMECs were lysed with lysis buffer containing 25 mM trizma hydrochloride, 150 mM sodium chloride, 0.5 mM ethylenediaminetetraacetic acid, 1% Triton-X 100, 1% sodium deoxycholate, 1mM dithiothreitol, 1 mM diisopropylfluorphosphat, 10 mM sodium fluoride and 250 μM sodium orthovanadate and protease inhibitor mix B (Sigma-Aldrich) on a spinning wheel for 30 min at 4°C. Protein GFP-Trap beads (Chromotek) (Rothbauer et al., 2008) were equilibrated with PBS/lysis buffer. Protein lysate was sonicated, centrifuged for 10 min at 13.000 rpm and 2500 μg protein lysate supernatant was added to beads and incubated for 2 h at 4°C. After washing with PBS/lysis buffer GFP-Trap beads were resuspended in 2× Lämmli sample buffer and boiled 10 min at 95°C. Western blot was performed using primary antibodies against Coro1B (Sigma-Aldrich) and Integrin linked kinase-1 (ILK1) (Cell Signaling Technology) and secondary IRDye 600 and 800 CW infra-red anti-mouse and anti-rabbit antibodies (LI-COR Biotechnology). Immune reactive bands were detected using the LICOR Infra-red reading system according to the manufacturer’s protocol.

### On-Bead Digestion and Mass Spectrometry

GFP-Trap beads (Chromotek) were incubated with protein lysate supernatants prepared as described above and processed with the iST Sample Preparation Kit (Preomics) according to the manufacturer’s protocol.

For LC-MS/MS purposes, desalted peptides were injected in an Ultimate 3000 RSLCnano system (Thermo Fisher Scientific) and separated using a 15-cm analytical column (75 μm ID home-packed with ReproSil-Pur C18-AQ 2.4 μm from Dr. Maisch) with a 50-min gradient from 5 to 60% acetonitrile in 0.1% formic acid. The effluent from the HPLC was directly electrosprayed into a Q_Exactive HF (Thermo Fisher Scientific) operated in data dependent mode to automatically switch between full scan MS and MS/MS acquisition. Survey full scan MS spectra (from *m*/*z* 375–1600) were acquired with resolution *R* = 60,000 at *m*/*z* 400 (AGC target of 3 × 106). The 10 most intense peptide ions with charge states between 2 and 5 were sequentially isolated to a target value of 1 × 10^5^, and fragmented at 27% normalized collision energy. Typical mass spectrometric conditions were: spray voltage, 1.5 kV; no sheath and auxiliary gas flow; heated capillary temperature, 250°C; ion selection threshold, 33.000 counts. MaxQuant 1.5.2.8 was used to identify proteins and quantify by iBAQ with the following parameters: Database, UP000005640_Hsapiens_170526; MS tol, 10 ppm; MS/MS tol, 10 ppm; Peptide FDR, 0.1; Protein FDR, 0.01 Min. peptide Length, five; Variable modifications, Oxidation (M); Fixed modifications, Carbamidomethyl (C); Peptides for protein quantitation, razor and unique; Min. peptides, 1; Min. ratio count, 2. Identified proteins were considered as interaction partners if their MaxQuant iBAQ values were greater than log2 two-fold enrichment and *p*-value 0.05 when compared to the control. Mass spectrometry data are available via ProteomeXchange with identifier PXD018947.

### Small Interfering RNA Transfection

HUVECs were transfected with small interfering RNA (siRNA) duplex against Coro1B (Sigma-Aldrich, SASI_Hs01_00084499 [siCoro1B #1] and SASI_Hs01_00084500 [siCoro1B #2]) and a scrambled control (Sigma-Aldrich, SIC001) using the MATra-si reagent (Promocell) and a magnet plate (Promocell) according to manufacturer’s protocol. Experiments were performed 72 h after transfection. All experiments were conducted with the two independent siRNA against Coro1B.

### Tube Formation in Matrigel

Matrigel basement membrane matrix (Corning) was plated on coverslips and incubated for 30 min at 37°C to allow polymerization. After treatment with siControl, siCoro1B #1 and siCoro1B #2 for 72 h, 85.000 HUVECs per mL were seeded on top of the matrigel matrix. Light microscopy images were taken with a 5× objective at an inverted laboratory microscope Leica DM IL LED. The total tube length and the number of master segments were analyzed with the ImageJ plugin angiogenesis analyzer.

### Statistics

Statistical analysis was performed using GraphPad Prism 6 (La Jolla, CA, United States). For pairwise comparison, the Student *t*-test, and for multiple comparisons, one-way ANOVA with the Dunnett’s method was applied. *P* < 0.05 were considered significant. Data are presented as the mean ± SD of at least three independent experiments.

## Results

### Coro1B Localizes to Cell–Cell Junctions in ECs

**FIGURE 1:**
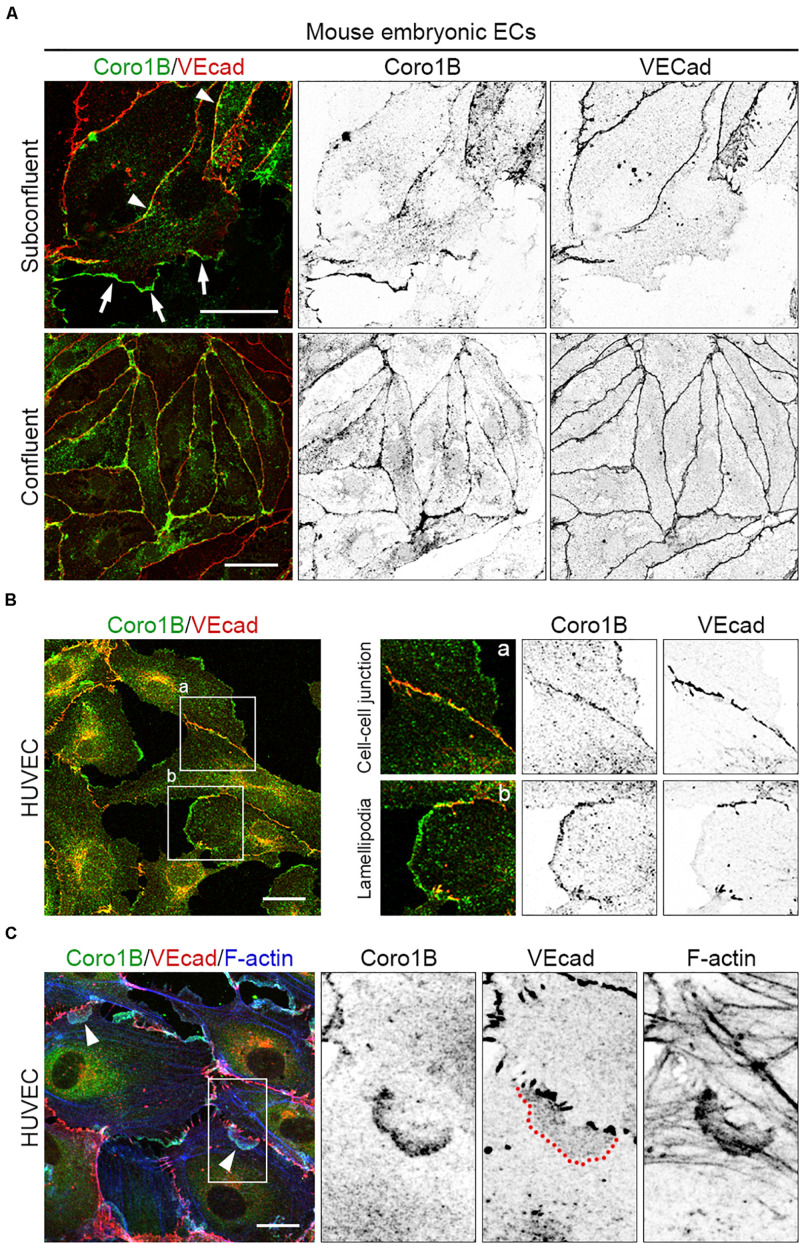
Coro1B localizes to endothelial cell–cell junctions. **(A)** Mouse embryonic ECs immunostained for Coro1B (green) and VEcad (red) under subconfluent and confluent conditions. Arrows point to localization of Coro1B at classical lamellipodia, arrowheads indicate Coro1B at cell–cell junctions. **(B)** HUVECs labeled with Coro1B (green) and VEcad (red). Localization of Coro1B at cell–cell junctions (a, zoom-in right, upper panel) and at classical lamellipodia (b, zoom-in right, lower panel). **(C)** Localization of Coro1B (green), VEcad (red) and F-actin (blue) in subconfluent HUVEC monolayers. Arrowheads point to localization of Coro1B and F-actin at the leading edge of JAIL. Red line indicates VEcad plaque appearance. Scale bars = 25 μm.

Coro1B regulates actin cytoskeleton organization at classical lamellipodia during cell spreading and migration (Mishima and Nishida, 1999; Chan et al., 2011). To study the role of Coro1B in actin cytoskeleton remodeling in ECs we first performed immunostaining of mouse and human primary ECs using specific antibodies against Coro1B and VEcad. As expected, the analysis showed that Coro1B localizes at the leading edge of classical lamellipodia in single cells as well as in subconfluent cell monolayers (Figures 1A,B). Interestingly, the analysis also revealed that a subset of Coro1B protein localizes close to cell–cell junctions where it partially colocalizes with VEcad (Figures 1A,B). In contrast to VEcad, Coro1B showed a discontinuous distribution pattern at cell–cell junctions (Figures 1A,B). This suggested that Coro1B is involved in actin cytoskeleton remodeling at cell–cell junctions. To investigate if Coro1B associates with F-actin at cell–cell junctions, we performed immunostaining on subconfluent HUVEC monolayers for Coro1B, VEcad and F-actin. The analysis showed that Coro1B is present at the leading edge of JAIL where it colocalizes with F-actin (Figure 1C). Immunofluorescent stainings in HMEC corroborate these findings (Supplementary Figure S1 in the Data Supplement). Together, these results demonstrated that Coro1B is present at cell–cell junctions of ECs where it localizes at leading edges of JAIL.

### Coro1B Is Dynamically Recruited to JAIL

**FIGURE 2:**
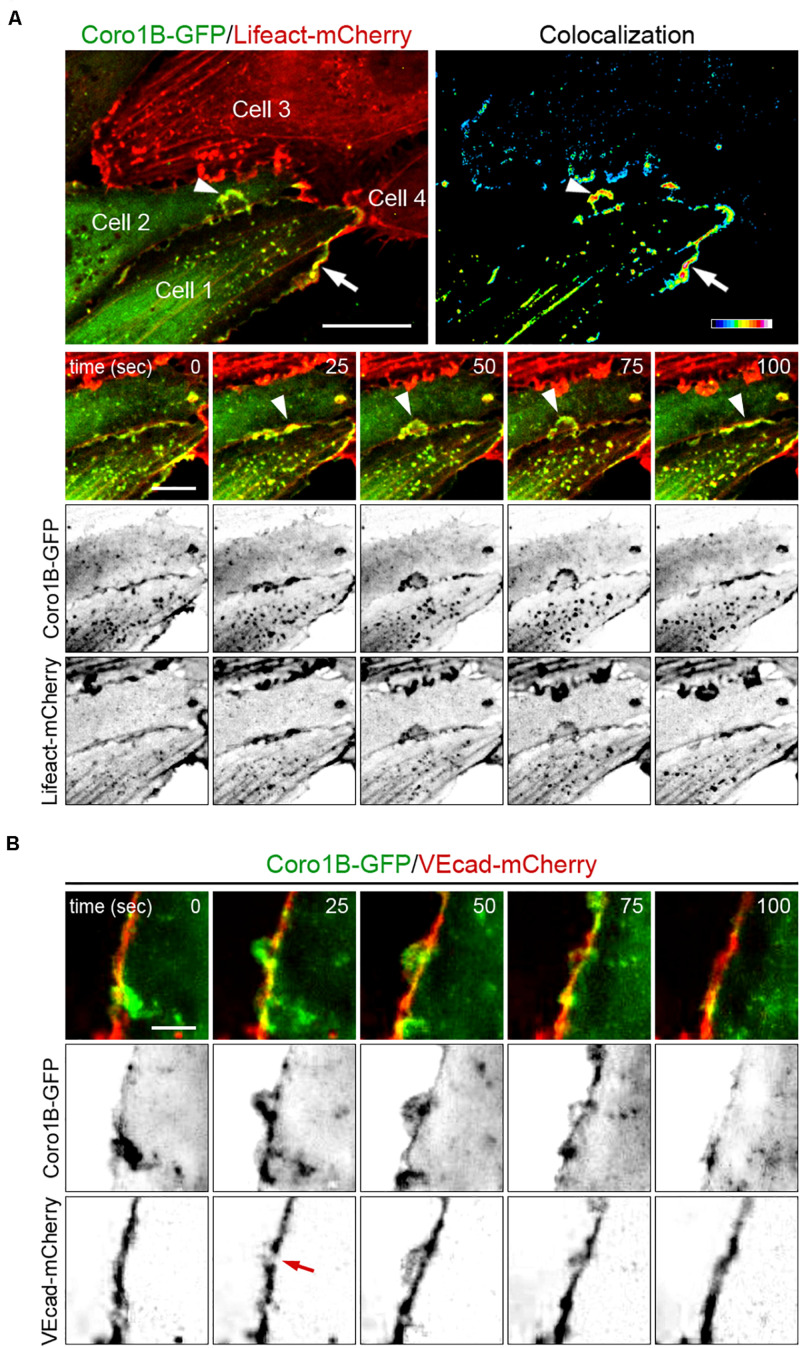
Dynamics of Coro1B, F-actin and VEcad at cell–cell junctions. Still images from time-lapse recording of HUVECs expressing **(A)** Lifeact-mCherry (red) and Coro1B-GFP (green), and **(B)** VEcad-mCherry (red) and Coro1B-GFP (green). **(A)** Localization of Coro1B-GFP and Lifeact-mCherry at the leading edge of lamellipodia (arrow) and JAIL (arrowheads) at indicated time points. Upper panel, right: Colocalization of Coro1B-GFP and Lifeact-mCherry is displayed in a heat map. Scale bar = 10 μm, color scale. Scale bar = 5 μm. **(B)** Localization of Coro1B-GFP (green) and VEcad-mCherry (red) in double transfected HUVECs during JAIL formation in still images at indicated time points. Red arrow points to local reduction of VEcad. Scale bar = 5 μm.

To assess Coro1B recruitment in relation to JAIL formation, we performed live imaging of HUVECs using lentiviral expression of Coro1B-GFP and Lifeact-mCherry. Imaging of subconfluent monolayers of these cells with spinning disk confocal microscopy revealed the highly dynamic occurrence of classical lamellipodia protrusions and JAIL at cell–cell junctions (Figure 2A; Video I in the Data Supplement). Coro1B was consistently recruited to the leading edge of classical lamellipodia protrusions and JAIL where it colocalizes with F-actin during the whole process of JAIL dynamics (Figure 2A). These findings suggest the involvement of Coro1B in the actin remodeling processes associated to JAIL formation. JAIL drive the dynamic rearrangement of VEcad at cell junctions, while maintaining monolayer integrity (Abu et al., 2014). Therefore, localization and dynamics of Coro1B and VEcad were evaluated in subconfluent monolayers of Coro1B-GFP and VEcad-mCherry expressing HUVECs (Figure 2B; Video II in the Data Supplement). The analysis showed that Coro1B protrusions appeared at the cell–cell junction in spots with a local reduction of VEcad expression (Figure 2B, 25 s). The Coro1B protrusion was followed by the formation of a VEcad plaque, which is the result of JAIL that overlap adjacent cells and lead directly to VEcad transactions in this area (Figure 2B, 50 s). Subsequently, the Coro1B protrusion regressed and VEcad showed a linear continuous distribution along the plasma membrane (Figure 2B, 100 s). JAIL formation was accompanied by the movement of the cell–cell junction (Supplementary Figure S2 in the Data Supplement).

### Localization of Coro1B at Cell–Cell Junctions Is Actin-Dependent

**FIGURE 3:**
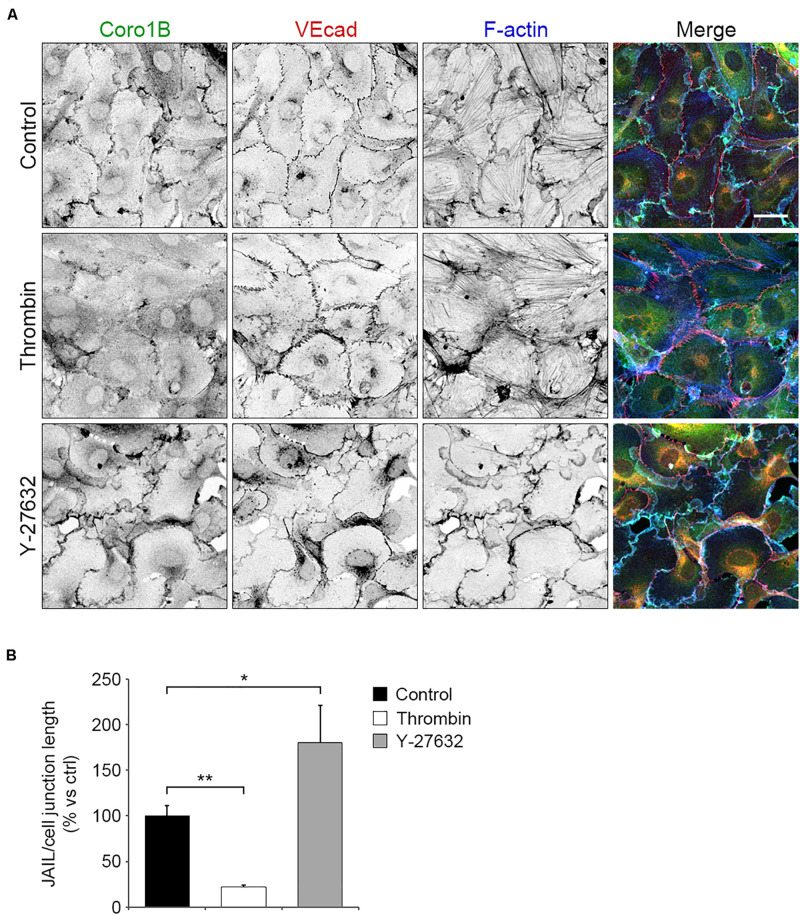
Coro1B localization at cell–cell junctions is regulated by actomyosin contraction. **(A)** HUVECs untreated (control) and treated with thrombin (0.2 U/ml) or Y-27632 (10 μM) and immunostained for Coro1B (green), VEcad (red) and F-actin (blue). Scale bar = 25 μm. **(B)** Quantitative analysis of JAIL number per cell–cell junction length in control and treated cells. Data represent mean ± SD. *P < 0.05, **P < 0.01. *n* = 3.

To understand how Coro1B is recruited to cell junctions, we investigated the formation of JAIL by manipulating actin cytoskeleton contraction. First, we used the endothelial permeability factor thrombin that induces actomyosin contraction and cell–cell junction disruption by activating RhoA (Wojciak-Stothard and Ridley, 2002). Treatment of subconfluent monolayers of HUVECs with thrombin for 10 min resulted in increased levels of radial actin bundles and discontinuous VEcad staining (Figure 3A). Additionally, quantification analysis revealed a strong significant reduction in JAIL number per cell–cell junction length in thrombin treated cells compared to control cells (Figure 3A). Interestingly, staining of Coro1B at the cell–cell junction was markedly reduced after thrombin stimulation (Figure 3A). In contrast, inhibition of actin cytoskeleton tension by using the Rho-kinase inhibitor Y-27632 resulted in continuous VEcad staining, increased levels of VEcad-associated cortical actin, a significant increase of the number of JAIL per cell junction field and a decidedly increased localization of Coro1B at cell–cell junctions (Figures 3A,B). Together, these data suggest that reduction of actomyosin contraction induces JAIL formation and Coro1B recruitment at cell–cell junctions.

### Coro1B Interacts With ILK and Colocalizes With α-pv in ECs

**FIGURE 4:**
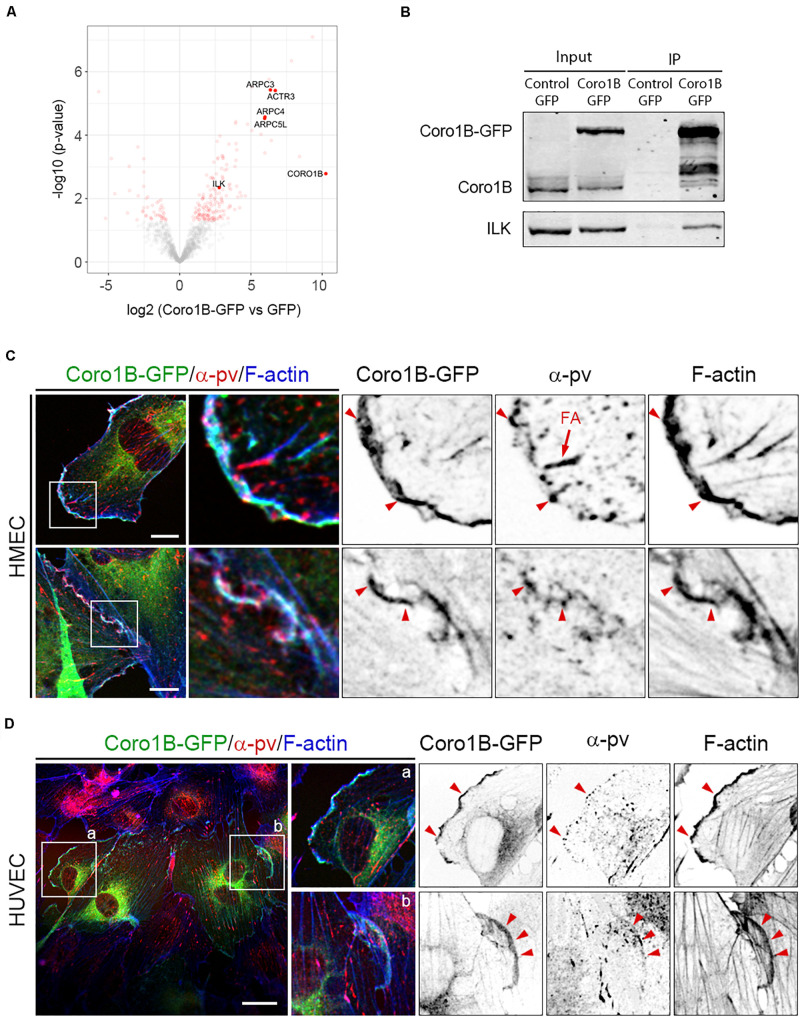
ILK is a new Coro1B-interacting protein. **(A)** Volcano plot showing the log2 fold enrichment of the proteins identified by label-free mass spectrometry in immunoprecipitates from Coro1B-GFP HMECs compared to the GFP control HMECs and the *p*-value (–log10) of the adjusted *t*-test comparing the abundance of these proteins in both immunoprecipitates. Proteins with a log2 fold enrichment above 2.0 and a *p*-value below 0.05 were considered significant (*n* = 4, see Supplementary Table S1). **(B)** Coro1B-GFP immunoprecipitation of ILK in Coro1B-GFP expressing HMECs. GFP expressing HMECs were used as a control. Whole-cell lysates are shown as the input. Coro1B-GFP expressing **(C)** HMECs and **(D)** HUVECs transfected with Coro1B-GFP (green) and stained for α-pv (red) and F-actin (blue). Arrowheads point to Coro1B-GFP,α-pv and F-actin colocalization. FA (arrow): focal adhesions. Scale bar = 25 μm.

To gain insights into the molecular mechanism of Coro1B recruitment to JAIL, we screened for interacting partners of Coro1B in ECs. To this end, we conducted immunoprecipitation experiments with HMECs expressing Coro1B-GFP or GFP alone followed by mass spectrometry analysis. Among the proteins that were precipitated with Coro1B we identified known Coro1B binding partners such as subunits of the Arp2/3 complex and cofilin as well as the novel interactor ILK (Figure 4A and Supplementary Table S1 in the Data Supplement) (Howell et al., 2015). Given that ILK has also been identified as a key regulator of EC function and blood vessel homeostasis, we further characterized the Coro1B-ILK interaction (Friedrich et al., 2004; Park et al., 2019). Consistently with the proteomic data, endogenous ILK was co-immunoprecipitated with Coro1B-GFP (Figure 4B). ILK forms a ternary complex (the IPP complex) with the adaptor proteins PINCH and α-pv, which stabilize each other and link the integrin-mediated cell-matrix adhesions to the actin cytoskeleton (Legate et al., 2006). As endothelial α-pv localizes at JAIL and is required for proper JAIL formation (Fraccaroli et al., 2015), we performed α-pv immunostaining in Coro1B-GFP expressing HMEC and HUVECs. The analysis showed that Coro1B colocalizes with α-pv at the leading edge of classical lamellipodia and JAIL (Figures 4C,D). These results support the hypothesis that JAIL formation involves Coro1B.

### Coro1B Controls Actin Cytoskeleton Organization and JAIL Formation

**FIGURE 5:**
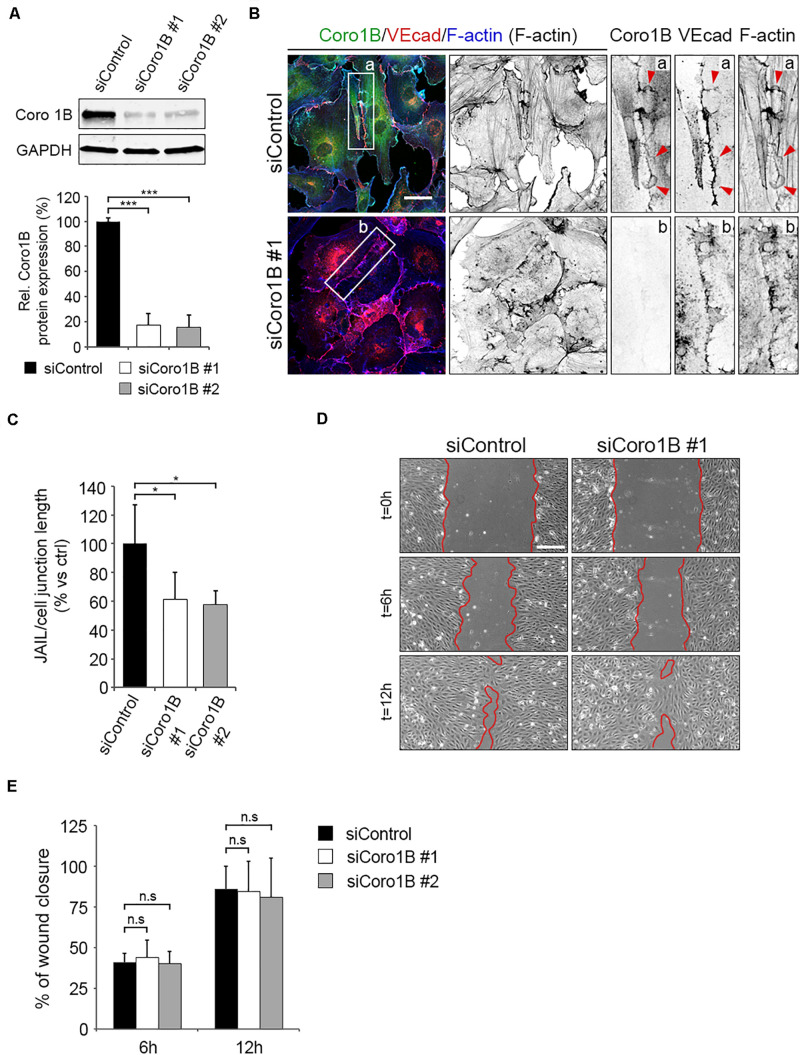
Depletion of Coro1B in ECs alters the organization of the actin cytoskeleton and the formation of JAIL. **(A)** Representative western blot analysis of Coro1B and GAPDH (loading control) in total lysates of control and Coro1B-depleted HUVECs. Graph shows three independent experiments. Values represent mean ± SD. ***P < 0.001. Data are from three independent experiments. **(B)** Control and Coro1B-depleted HUVEC immunostained for Coro1B (green), VEcad (red) and F-actin (blue). Zoom-in indicate presence (a) or absence (b) of Coro1B at cell–cell junctions. Arrowheads point to JAIL. Scale bar = 25 μm. **(C)** Quantitative analysis of JAIL number per cell–cell junction length in control and Coro1B-depleted HUVECs. Values are normalized to control. Data represent mean ± SD. *P < 0.05, *n* = 4. **(D)** Representative phase-contrast images of control and Coro1B-depleted HUVECs in a scratch-wound assay (*t* = 0, *t* = 6, and *t* = 12 h after scratch). Red lines highlight the unclosed wound area. Scale bar = 200 μm. **(E)** Graph showing the mean ± SD percentage of wound closure of control and Coro1B-depleted HUVECs at two time points during scratch-wound assay. Data are from three independent experiments. ns; not significant.

To decipher the functional relevance of Coro1B for JAIL formation in ECs, we depleted Coro1B expression through siRNAs. Two of the siRNA tested induced efficient significant knockdown of Coro1B protein levels in HUVECs (Figures 5A,B). To study the role of Coro1B on the actin cytoskeleton and JAIL formation, we immunostained subconfluent monolayers of control and Coro1B-depleted HUVECs for Coro1B, VEcad and F-actin. Coro1B knockdown resulted in reduced stress fibers, discontinuous cortical actin, and disorganized VEcad compared to control cells (Figure 5B). Quantification analysis showed a significant decrease in number of JAIL per cell–cell junction length in Coro1B-depleted cells when compared to control cells (Figure 5C). We next examined whether endothelial Coro1B is involved in collective cell migration. To do this, we performed scratch wound assays and found no differences in cell migration between control and Coro1B-depleted cells (Figures 5D,E). Together, these results indicated that Coro1B controls actin cytoskeleton rearrangement and JAIL formation in ECs but it is dispensable for collective EC migration.

### Coro1B Is Critical for Endothelial Network Assembly *in vitro*

**FIGURE 6:**
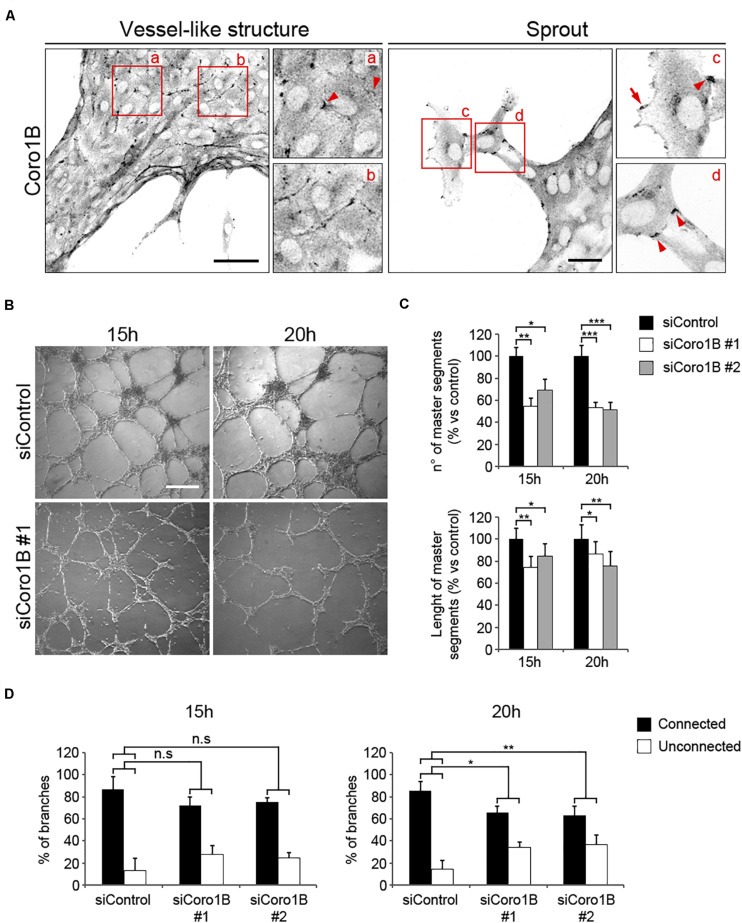
Coro1B is required for proper endothelial network assembly in matrigel. **(A)** HUVECs cultured on matrigel for 20 h and immunostained for Coro1B. Scale bar = 50 μm (left) and 25 μm (right). Arrowheads point to Coro1B localization at JAIL-like structures and arrow indicates Coro1B localization at classical lamellipodium. **(B)** Representative phase-contrast images of tube networks of control and Coro1B-depleted HUVECs at indicated time points after seeding. Scale bar = 200 μm. Quantitative analysis of **(C)** the number and length of master segments and **(D)** connected and unconnected tube networks of control and Coro1B-depleted HUVECs. Values are normalized to control. Data represent mean ± SD of three independent experiments. *P < 0.05, **P < 0.01, ***P < 0.001. ns; not significant.

It has been recently shown that JAIL are essential for blood vessel formation and vascular homeostasis (Cao et al., 2017). To establish whether Coro1B plays a role in vessel formation, control and Coro1B-depleted HUVECs were cultured on matrigel coats and tube formation was assessed as previously described (Montanez et al., 2002). First, we performed immunostaining of control vessel-like structures with Coro1B. The analysis showed that Coro1B localizes at putative JAIL at cell junctions (Figure 6A, Box a,b). In vessel sprouts Coro1B was localized at classical lamellipodia (Figure 6A, Box c) and cell–cell junctions (Figure 6A, Box c,d). To address the functional role of Coro1B for EC tube formation, we performed the matrigel assay using HUVECs after treatment with siRNA against Coro1B or control siRNA and subsequently analyzed tube formation. The analysis showed that depletion of Coro1B significantly reduced the number of master segments as well as the length of master segments compared to control conditions (Figures 6B,C). Furthermore, the percentage of connected branches was significantly diminished in Coro1B-depleted cells (Figure 6D). These results indicate that Coro1B is critically required for proper *in vitro* tube network formation.

## Discussion

In this study we identified the actin-binding protein Coro1B as a novel component and regulator of endothelial cell–cell junctions. Our data show that Coro1B is recruited to, and operates at, actin-driven protrusions at cell–cell junctions called JAIL. The recruitment of Coro1B to cell–cell junctions and the formation of JAIL are regulated by actin cytoskeleton remodeling and contraction. Using mass spectrometry, we identified ILK as a new Coro1B-interacting protein in ECs. Finally, depletion of Coro1B in ECs leads to defects in actin cytoskeleton organization, reduced number of JAIL, altered cell–cell junction morphology and impaired endothelial network assembly.

The actin cytoskeleton enables many dynamic cellular activities, including lamellipodia protrusion, cell migration and cell–cell junction formation and maintenance (Edwards et al., 2014). To do this, the actin filaments undergo continuous cycles of polymerization and depolymerization regulated by actin-binding proteins including the Arp2/3 complex, cofilin and coronins, and are often induced in response to extracellular signals. The actin polymerizing proteins of the Arp2/3 complex regulate migration of EC and integrity of endothelial cell–cell junctions, thereby being essential for angiogenesis (De Smet et al., 2009; Garcia-Ponce et al., 2015; Phng et al., 2015). Recently it has been shown that JAIL, actin-driven and Arp2/3 complex-controlled plasma membrane protrusions that develop at cell–cell junction sites with decreased or lost VEcad, regulate the local dynamics and patterning of VEcad, thereby controlling junctional integrity and monolayer formation *in vitro* and sprouting angiogenesis *in vivo* (Fraccaroli et al., 2015; Cao et al., 2017). Our results showed that in addition to classical lamellipodia, Coro1B is found at cell–cell junctions and at the leading edge of JAIL in ECs, suggesting that Coro1B regulates actin cytoskeleton dynamics at cell–cell junctions. Cytoskeleton manipulation experiments showed that the mechanisms underlying Coro1B recruitment to cell–cell junctions involve actin cytoskeleton tension. While thrombin-stimulated actomyosin contraction reduces Coro1B localization at the cell–cell junction and JAIL formation, inhibition of actomyosin contraction with the Rho-kinase inhibitor Y-27632 increases Coro1B localization at the cell–cell junctions and JAIL formation. These findings suggest a functional interaction between Coro1B, the actin cytoskeleton and JAIL, which is in agreement with previous reports (Cao et al., 2017). Quantification analysis of JAIL in control and Coro1B-depleted ECs clearly show that once Coro1B is functionally perturbed, the frequency of JAIL is reduced. This reduction in JAIL formation is associated with disorganized actin cytoskeleton and discontinuous distribution of VEcad at cell–cell junctions. In migrating fibroblasts, Coro1B induced the dissociation of the Arp2/3 complex from actin filaments, which is critical for classical lamellipodia formation, thereby promoting the disassembly of the actin network in lamellipodia (Cai et al., 2008). By the same mechanism, Coro1B could regulate JAIL formation at cell–cell junctions in ECs. In epithelial cells, Coro1B was shown to regulate actin cytoskeleton reorganization and cell–cell junction stability through RhoA signaling (Michael et al., 2016; Priya et al., 2016). Together with our experiments, this data point to an essential role of Coro1B in regulating endothelial and epithelial cell–cell junctions.

To understand how Coro1B localizes to cell–cell junctions and JAIL, we performed pulldown experiments followed by mass spectrometry analysis. The finding of well-known Coro1B binding proteins such as several subunits of the Arp2/3 complex among the top hits in our interactome screen validates our results. Although our immunostaining studies show partial colocalization of Coro1B and the VEcad-catenin complex at cell–cell junctions, we did not find VEcad or α- or β-catenin in our interactome suggesting no direct binding between Coro1B and the VEcad-catenin complex. Our analysis identified ILK as a new Coro1B-interacting protein. ILK is known to bind to α-pv and PINCH to form the IPP-complex, which critically controls integrin signaling at focal complexes and focal adhesions (Legate et al., 2006; Wickstrom et al., 2010). ILK is essential for vessel development and vessel homeostasis (Friedrich et al., 2004; Tan et al., 2004; Park et al., 2019). Moreover, ILK regulates cell–cell junctions in epithelial cells (Vespa et al., 2005). In addition to its localization to integrin-mediated adhesions, α-pv is recruited to JAIL in ECs, where it regulates actin rearrangement and VEcad organization (Fraccaroli et al., 2015). As such, α-pv controls sprouting angiogenesis and blood vessel stability (Fraccaroli et al., 2015). Our immunostaining studies show that Coro1B is not present in the focal adhesions and suggest that Coro1B-ILK interaction takes place in the focal complexes at the leading edge of classical lamellipodia. The analysis also demonstrates a colocalization of Coro1B and α-pv at JAIL, suggesting that they cooperate at JAIL to control F-actin remodeling.

Our functional analysis data shows that Coro1B is not essential for collective migration of ECs. However, JAIL-mediated cell–cell junction remodeling is critical for sprouting angiogenesis and blood vessel network formation in mice (Cao et al., 2017). To decipher whether Coro1B is needed for vessel network assembling, we conducted tube network formation assays on matrigel, a matrix rich in ECM components such as laminin and collagen-IV. The depletion of Coro1B reduces the number of tubes, their length and the complexity of the network. Moreover, the number of unconnected tubes increases over time in Coro1B-depleted cells compared to control cells, suggesting that Coro1B is needed for tube stability. Taken together, our findings suggest that Coro1B is required for endothelial cell–cell junction remodeling and blood vessel network formation/maintenance. New experiments are now needed to determine the role of Coro1B on sprouting angiogenesis, endothelial barrier and blood vessel permeability *in vivo*.

## Data Availability Statement

All datasets presented in this study are included in the article/Supplementary Material.

## Author Contributions

H-JS, BW, and EM designed the experiments. A-CW, LW, MS, BP, JC, and DM-B performed the experiments. IF, H-JS, BW, and EM interpreted the results. A-CW, LW, and EM wrote the manuscript. All authors contributed to the article and approved the submitted version.

## Conflict of Interest

The authors declare that the research was conducted in the absence of any commercial or financial relationships that could be construed as a potential conflict of interest.
