# Supplementary material for: Coronin 1B Controls Endothelial Actin Dynamics at Cell–Cell Junctions and Is Required for Endothelial Network Assembly
Source: Front Cell Dev Biol. 2020 Jul 31;8:708. doi: 10.3389/fcell.2020.00708 (PMC7411154; doi:10.3389/fcell.2020.00708)
Supplement: Supplementary file 1 [file Data_Sheet_1.pdf]

DATA SUPPLEMENT

Coronin 1B controls endothelial actin dynamics at cell-cell junctions and is required for endothelial network assembly

Ann-Cathrin Werner[1,2,*], Ludwig T Weckbach[1,2,3,*], Melanie Salvermoser[1,2], Bettina Pitter[1,2], Jiahui Cao[4], Daniela Maier-Begandt[1,2], Ignasi Forné[5], Hans-Joachim Schnittler[4], Barbara Walzog[1,2,†] and Eloi Montanez[1,2,6,†]

[1] Institute of Cardiovascular Physiology and Pathophysiology, Biomedical Center, LMU Munich, 82152 Planegg-Martinsried, Germany
[2] Walter Brendel Center of Experimental Medicine, University Hospital, LMU Munich, 81377 Munich, Germany
[3] Medizinische Klinik I, Klinikum Großhadern, 81377 Munich, Germany
[4] Institute of Anatomy and Vascular Biology, Westfälische Wilhelms-Universität Münster, 48149 Münster, Germany
[5] Protein Analysis Unit, Biomedical Center, LMU Munich, 82152 Planegg-Martinsried, Germany
[6] Department of Physiological Sciences, Faculty of Medicine and Health Sciences, University of Barcelona and IDIBELL, Barcelona, Spain

Figure I: Coro1B localizes to cell-cell junctions in HMECs. HMECs immunostained for Coro1B (green), VEcad (red) and F-actin (blue) show localization of Coro1B at the leading edge of classical lamellipodia (a, zoom-in upper panel), at the leading edge of JAIL and cell-cell junctions (b, zoom-in lower panel). Scale bars = 25 µm.

Figure II: Dynamic of cell-cell junction over time. Representative microscopic still images of HUVECs transfected with Coro1B-GFP (green) and VEcad-mCherry (red) illustrating the movement of the cell-cell junction at indicated time points. Scale bar = 10 µm.

Video I: Dynamics of Coro1B and F-actin at cell-cell junctions. Live imaging of HUVECs expressing of Coro1B-GFP and Lifeact-mCherry. Scale bars = 10 µm.

Video II: Dynamics of Coro1B and VEcad at cell-cell junctions. Live imaging of HUVECs expressing of Coro1B-GFP and VEcad-mCherry. Scale bars = 10 µm.

Table I: Coro1B interactome in ECs. Coro1B interacting proteins were identified by LC-MS analysis. Resulting raw data were analyzed using MaxQuant software. Gene names of the identified proteins are based on the UniProt database. The gene name correlates with the Gene Name from figure 4. Displayed are all proteins with a log2 fold change above 2 and p-value below 0.05. n = 4.

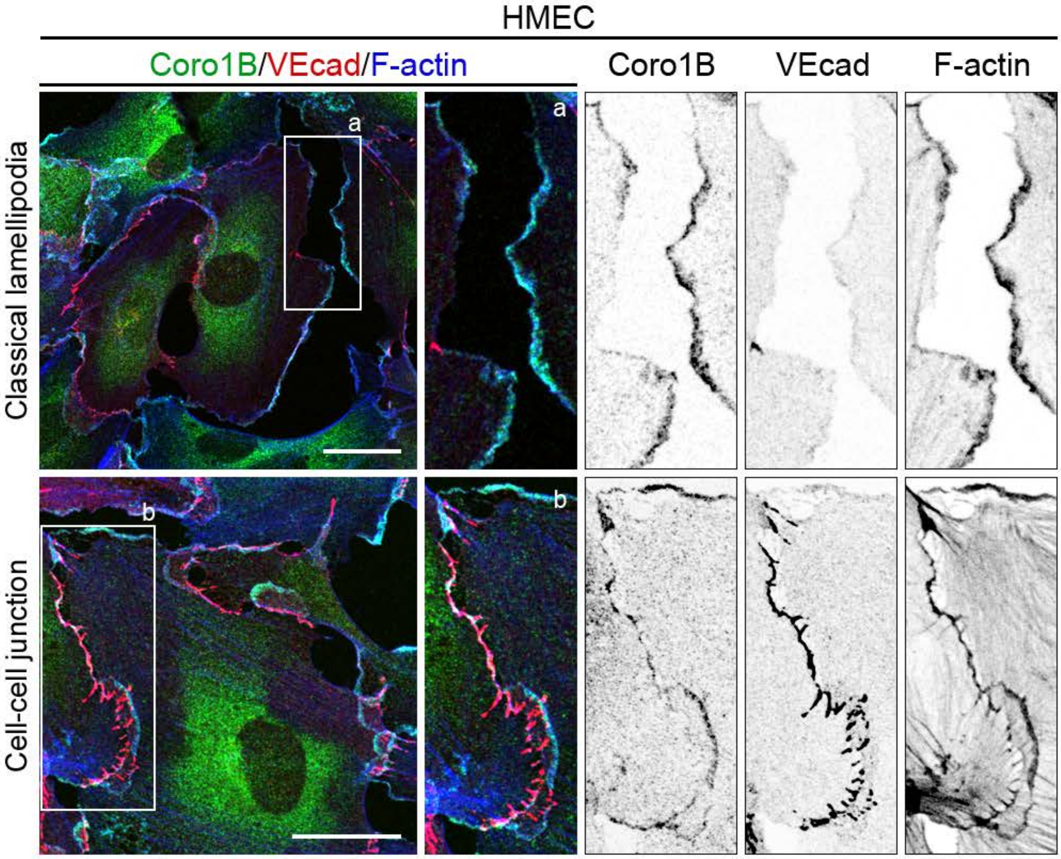
HMEC
Coro1B/VEcad/F-actin
Coro1B
VEcad
F-actin
Classical lamellipodia
Cell-cell junction
a
a
b
b

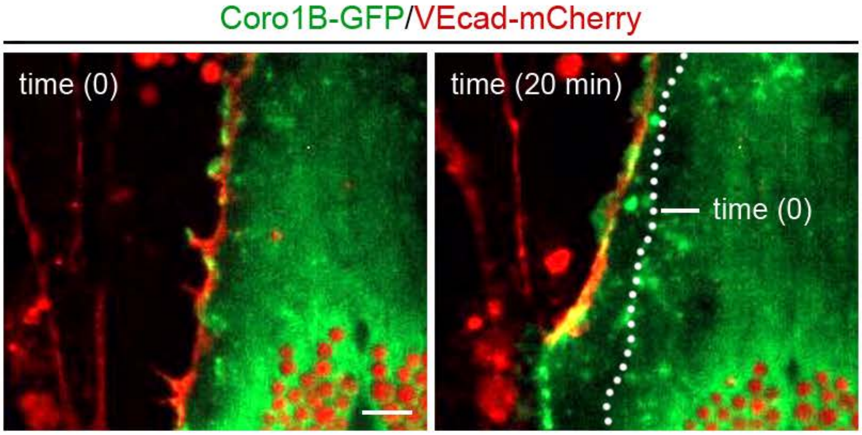
Coro1B-GFP/VEcad-mCherry
time (0)
time (20 min)
time (0)
